# Karyological and nuclear DNA content variation of the genus *Asparagus*

**DOI:** 10.1371/journal.pone.0265405

**Published:** 2022-03-16

**Authors:** Susann Plath, Evelyn Klocke, Thomas Nothnagel

**Affiliations:** Julius Kühn Institute (JKI)—Federal Research Centre for Cultivated Plants, Institute for Breeding Research on Horticultural Crops, Quedlinburg, Germany; Leibniz-Institute of Plant Genetics and Crop Plant Research (IPK), GERMANY

## Abstract

*Asparagus* wild relatives could be a promising possibility to extent the genetic variability of garden asparagus and for new cultivars with favorable traits such as high yield stability, disease resistance and stress tolerance. In order to achieve an efficient use in breeding, a detailed cytogenetic characterization of the accessions is necessary. This study worked on 35 *Asparagus* accessions, including *A*. *officinalis* cultivars (‘Darlise’, ‘Ravel’ and ‘Steiners Violetta’) and *Asparagus* wild relatives, for which the number of chromosomes, their size, the nuclear DNA content, and the genomic distribution of 5S and 45S rDNA were analyzed. Different ploidy levels (diploid, triploid, tetraploid, pentaploid and hexaploid) were found. Furthermore, the size of the chromosomes of all diploid *Asparagus* accessions was determined which led to differences in the karyotypic formula. *A*. *plocamoides* harbors the smallest chromosome with 1.21 μm, whereas the largest chromosome with 5.43 μm was found in *A*. *officinalis*. In all accessions one 5S rDNA locus per genome was observed, while the number of 45S rDNA loci varied between one (*A*. *albus*, *A*. *plumosus*, *A*. *stipularis*) to four (*A*. *setaceus*). In most *Asparagus* accessions, the 5S and 45S rDNA signals were located on different chromosomes. In contrast, the genomes of *A*. *africanus*, *A*. *plocamoides*, *A*. *sp*. (a taxonomically unclassified *Asparagus* species from Asia) and *A*. *verticillatus* (diploid accessions) have one 5S and one 45S rDNA signal on the same chromosome. The measured 2C DNA content ranges from 1.43 pg (*A*. *plocamoides*, diploid) to 8.24 pg (*A*. *amarus*, hexaploid). Intraspecific variations for chromosome number, karyotypic formula, signal pattern with 5S and 45s rDNA probes and DNA content were observed. Interspecific variations were also recognized in the genus *Asparagus*.

## Introduction

The genus *Asparagus* has its primary diversification hotspot in southern Africa and contains more than 210 species which are distributed in temperate and tropical regions [1]. Different species of the *Asparagus* genus species have an economical value. The most prevalent kind is *A*. *officinalis* L. (garden asparagus) which is cultivated worldwide as a vegetable crop. In Africa, Asia and the Mediterranean countries it is common to collect for self-consumption young shoots of some wild Asparagus spp. (*A*. *acutifolius* L., *A*. *aphyllus* L., *A*. *acerosus* Thunb. ex Schult. & Schult. f., *A*. *laricinus* Burch. among other). *A*. *verticillatus* L. is used for medicinal purposes while *A*. *densiflorus* (Kunth) Jessop and *A*. *plumosus* Baker serve as ornamentals [2]. The most prevalent kind is *A*. *officinalis* (garden asparagus) which is cultivated worldwide as a vegetable [3]. Due to its perennial growth, garden asparagus can be infected with several diseases causing a decrease of production (asparagus decline). The most common virus infections are *Asparagus virus 1* (AV-1), *Asparagus virus 2* (AV-2) and *Cucumber mosaic virus* (CMV) [4]. Additional infestation caused by *Fusarium* spp., *Puccinia asparagi*, *Stemphylium vesicaria* and *Phytophthora* spp. causes high economic damages [1, 5, 6]. These diseases cannot be durable controlled by plant protection products; consequently, breeding resistant cultivars is the most feasible solution.

In contrast, extensive investigations have revealed that wild *Asparagus* species are resistant to several diseases. Studies show that *A*. *stipularis* Forssk is resistant to rust (*Puccinia asparagi*) but is susceptible to *Fusarium* [7, 8]. *A*. *densiflorus* is resistant to a variety of diseases, for example *Fusarium* spp., *Puccinia asparagi* and *Stemphylium vesicarium* [9–12]. Additionally, 29 different wild accessions resistant to AV-1 have been identified [13]. Furthermore, it is known that *Asparagus* wild relatives have also many desirable agricultural traits against abiotic stress such as salt and drought tolerance [14]. The introgression of these desirable traits is a very important goal in asparagus pre-breeding programs. Several crosses between garden asparagus and *Asparagus* wild relatives have been carried out. Crosses between *A*. *officinalis* x *A*. *amarus* DC., *A*. *officinalis* x *A*. *maritimus* (L.) Mill., *A*. *officinalis* x *A*. *prostrates* Dumort. and *A*. *officinalis* x *A*. *pseudoscaber* Grecescu are examples for the creation of interspecific hybrids [15–17]. Indeed, several attempts to cross *A*. *officinalis* with *A*. *acutifolius*, *A*. *densiflorus*, *A*. *stipularis* or *A*. *verticillatus* have not been successful yet [16–20]. In contrast, crosses between closely related species at the same ploidy level have mostly been successful [21]. Until now, the cytogenetic knowledge about wild *Asparagus* species is limited. To use these accessions for breeding a detailed characterization is necessary.

The genus *Asparagus* contains three subgenera *Asparagus*, *Protasparagus* and *Myrsiphyllum* [22]. In the last few years, several molecular analyses have been carried out to get a better understanding of the phylogenetic relationships of the *Asparagus* species. Studies using restriction fragment length polymorphism (RFLP), or simple sequence repeats (SSR) divided the European and African species into two monophyletic groups [3, 13].

Due to limited research, mostly focused on *A*. *officinalis*, cytogenetic findings about the *Asparagus* genus are restricted in numbers. It is known that the basic chromosome number of *Asparagus* is ten (x = 10), *A*. *officinalis* is diploid, and the size of a haploid genome is 1.308 Mbp/1C [23]. For some wild species, the number of chromosomes and their ploidy levels were reported, ranging from diploid to dodecaploid [1, 3, 21, 24, 25]. However, many chromosome analyses are incomplete or unavailable for several wild *Asparagus* species. As a result, the common knowledge of the chromosome organization of *Asparagus* wild relatives is extremely limited.

The aim of this research was to characterize a set of 32 wild *Asparagus* species to increase the knowledge about cytogenetic diversity between itself and the garden asparagus. So, with a better understanding of the taxonomic relationships, suitable wild *Asparagus* species can then be included in breeding programs.

## Material and methods

### Plant material

Plants of 32 *Asparagus* species and three cultivars of *A*. *officinalis* are analyzed in this work. The origin of the seeds or plants as well as the taxonomic classification is given in Table 1. Seeds were sown in a sand-humus mixture (3:1 v/v) and plantlets were cultivated in plastic pots under greenhouse conditions.

**Table 1:** *Asparagus* accessions used for the study, their ploidy level and the estimated average genome size (2C DNA content).

Accession	Subgenus^1^	Origin	Seed Origin^2^	Ploidy Level^3^	IDS^4^	n^5^	2C DNA-Content		
							Mean (pg) ± SD	Mbp^6^	CV (%)^7^
*A*. *acutifolius* L.	A	Italy, Vittoria	CRA	2n = 2x = 20	S	9	2.70 ± 0.10	2641	3.58
*A*. *aethiopicus* L.	P	Malaga	BDA	2n = 6x = 60	S	7	5.17 ± 0.23	5056	4.51
*A*. *africanus* Lam.	A	Australia	AUS	2n = 2x = 20	P	3	2.10 ± 0.02	2057	0.99
*A*. *albus* L.	A	Portugal	EVO	2n = 2x = 20	S	6	2.46 ± 0.10	2400	4.08
*A*. *amarus* DC.	A	Italy	CRA	2n = 6x = 60	S	5	8.24 ± 0.20	8063	2.38
*A*. arborescens*	A	Canary	GRU	2n = 2x = 20	S	3	2.63 ± 0.04	2572	1.37
*A*. *densiflorus* (Kunth) Jessop 1	P	Israel	ISR	2n = 6x = 60	S	8	5.04 ± 0.14	4927	2.84
*A*. *densiflorus* (Kunth) Jessop 2	P	Israel	ISR	2n = 6x = 60	S	4	5.09 ± 0.13	4973	2.51
*A*. *densiflorus* (Kunth) Jessop 3	P	Ibiza	IBZ	2n = 4x = 40	S	7	3.57 ± 0.11	3489	2.69
*A*. *maritimus* (L.) Mill. 1	A	Italy	BGH	2n = 6x = 60	S	4	7.98 ± 0.04	7804	0.53
*A*. *maritimus* (L.) Mill. 2	A	Italy	VRE	2n = 6x = 60	S	4	7.78 ± 0.11	7612	1.37
*A*. *maritimus* (L.) Mill. 3	A	Italy, Mavar.	CRA	2n = 6x = 60	S	5	7.69 ± 0.23	7525	3.00
*A*. *maritimus* (L.) Mill. 4	A	Italy, Vign.3	CRA	2n = 6x = 60	S	3	7.76 ± 0.12	7540	1.56
*A*. *officinalis* L. ‘Darlise’	A	France	DAR	2n = 2x = 20	S	14	2.95 ± 0.07	2882	2.50
*A*. *officinalis* L. ‘Ravel’	A	Germany	SWS	2n = 2x = 20	P	11	3.04 ± 0.04	2982	1.35
	A				S	3	3.06 ± 0.02	2993	0.57
*A*. *officinalis* L. ‘Steiners Violetta’	A	Germany	GAT	2n = 4x = 40	S	3	6.37 ± 0.04	6227	0.55
*A*. *pastorianus* Webb & Berthel.	A	Macaronesia	GRU	2n = 4x = 40	S	3	5.59 ± 0.04	5467	0.78
*A*. *plocamoides* Webb ex Svent.	A	Canaries	GRU	2n = 2x = 20	S	4	1.43 ± 0.03	1394	2.81
*A*. *plumosus* Baker	A	Cuba	HAV	2n = 2x = 20	P	3	2.11 ± 0.02	2064	0.82
*A*. *prostratus* Dumort. 1	A	France, Ploemever	VIL	2n = 4x = 40	S	4	5.92 ± 0.09	5792	1.57
*A*. *prostratus* Dumort. 2	A	France, Gavres	VIL	2n = 4x = 40	S	3	6.14 ± 0.04	6002	0.62
*A*. *prostratus* Dumort. 3	A	France, Damgan	VIL	2n = 4x = 40	S	5	6.05 ± 0.04	5921	0.71
*A*. *prostratus* Dumort. 4	A	France, Houat Is.	VIL	2n = 4x = 40	S	11	6.16 ± 0.08	6026	1.23
*A*. *pseudoscaber* Grecescu	A	Italy	LIM	2n = 6x = 60	S	3	7.70 ± 0.05	7531	0.60
*A*. *ramosissimus* Baker	M	Angola	GRU	2n = 2x = 20	S	4	2.31 ± 0.01	2254	0.25
*A*. *scoparius* Lowe	A	Africa, Cape Verde	GRU	2n = 2x = 20	S	3	1.46 ± 0.01	1425	0.40
*A*. *setaceus* (Kunth) Jessop	A	SE-Africa	GRU	2n = 4x = 40	S	3	2.96 ± 0.04	2898	1.19
*A*. *stipularis* Forssk. 1	A	Ibiza	IBZ	2n = 2x = 20	S	3	1.62 ± 0.02	1588	0.94
*A*. *stipularis* Forssk. 2	A	Ibiza	IBZ	2n = 2x = 20	S	4	2.26 ± 0.03	2213	1.10
*A*. *stipularis* Forssk. 3	A	Ibiza	IBZ	2n = 2x = 20	S	4	2.42 ± 0.01	2369	0.4
*A*. *stipularis* Forssk. 4	A	Cyprus	LIM	2n = 2x = 20	S	3	2.37 ± 0.08	2321	3.51
*A*. *verticillatus* L. 1	A	?	BGH	2n = 4x = 40	S	3	6.08 ± 0.01	5946	0.16
*A*. *verticillatus* L. 2	A	? ASP 5	GAT	2n = 2x = 20	S	3	3.36 ± 0.02	3221	0.63
*A*. *verticillatus* L. 3	A	?	LIM	2n = 2x = 20	S	4	3.08 ± 0.02	3015	0.77
*A*. *sp*. **	(P)	Asia	BVR	2n = 2x = 20	P	6	1.86 ± 0.03	1822	1.72

* Willd. Ex Schult. & Schult.f.;

** not taxonomically classified;

^1^ Subgenera: A–*Asparagus*, M–*Myrsiphyllum*, P–*Protasparagus* [1, 22], (‥) preliminary own classicfication,

^2^ AUS–invasive weed in Queensland (Australia), BGD–Botanical Garden Berlin Dahlem (Germany), BGH–Botanical Garden Hamburg (Germany), BVR–Beijing Vegetable Research Center (China), CRA–Research Institute for Vegetable Crops, Montanaso Lombardo (Italy), DAR–Darbonne/Inotalis (France), EVO–University of Evora (Portugal), GAT- Institute of Plant Genetics and Crop Plant Research Gatersleben (Germany), GRU–Botanical collection of Gruson-Gewächshäuser Magdeburg (Germany), HAV–displaced invasive weed on Cuba, Havanna (Cuba), IBZ–ornamental and wild asparagus from Ibiza (Spain), ISR–Volcani Centre Bet Dagan (Israel), LIM–Limgroup B.V. Horst (The Netherlands), SWS–Süd-West Saat, Rastatt (Germany), VIL–Vilmorin (France), VRE–Vreekens´Zaden, Dordrecht (The Netherlands);

^3^ Classified by chromosome counting for all wild relatives used in this study,

^4^ IDS–internal DNA Standard: S—*Solanum lycopersicon* ‘Stupicke’, 1.96 pg DNA; P—*Pisum sativum* ‘Ctirad’, 9.09 pg DNA;

^5^ n –Number of analyzes,

^6^ Conversion factor 1 pg = 978 Mbp (Doležel *et al*. 2003) [33] ^,^

^7^ CV–Coefficient of variation of relative DNA content was calculated as (SD/Mean) x 100 (%).

### Chromosome preparation and chromosome counting

For mitotic metaphase spreads actively growing young roots and spears were collected in distilled water and placed on ice +for 1–1.5 h. The meristem tissue was treated with 2 mM 8-hydroxyquinoline for 2.5 h, followed by fixation in freshly prepared 3:1 ethanol: acetic acid over night at room temperature and then stored at 4°C. Meristems have been rinsed with distilled water for 10 min and then macerated in an enzyme mixture containing 4% cellulase (´Onozuka R-10´, Duchefa Biochemie, Haarlem, The Netherlands) and 1% pectolyase Y-23 (Seishin Pharmaceutical Co., Tokyo, Japan) in 75 mM KCl and 7.5 mM Na_2_-EDTA adjusted to pH 4.0 [26]. Enzymatic digestion was carried out at 37°C for 40 to 50 min. The macerated meristems have been washed in distilled water for at least 10 min and squashed in 45% acetic acid. The chromosomes were counted under phase-contrast using an Axioskop 2 microscope (Zeiss, Oberkochen, Germany). Images were captured using an AxioCam MRm (Zeiss, Oberkochen, Germany) camera. For the determination of the chromosome number (2n), more than five metaphase cells from at least two individual root tips were analyzed.

The chromosome lengths of diploid *Asparagus* accessions were measured in the individual metaphase plate using the application Axio manager (Zeiss, Oberkochen, Germany) and were then sorted according to size. The mean value of the sorted chromosomes was determined and divided into three chromosome size groups. Chromosomes smaller than 3 μm were classified as small (S). Between 3 μm and 4 μm large chromosomes were assigned as medium-sized (M) chromosomes. In the large-sized (L) group, the chromosomes were classified larger than 4 μm. To determine the karyotype, between ten and thirty-two metaphase cells from at least three different individual root tips were examined. The data are shown in S1 Table.

### Fluorescence *in situ* hybridization (FISH)

Total genomic DNA from *A*. *officinalis* ‘Eposs’ was extracted from 50 mg young phylloclades [27] and used for amplification of the rDNA 5S and 45S probes. Amplification was carried out in 25 μl reaction mix containing 20 ng plant genomic DNA, 10 x Dream Taq Puffer, 2 mM dNTP´s Mix with dATP, dGTP, dCTP, dTTP, 0,5 μm of each primer and 1 unit of Dream Taq polymerase (Thermo Fisher Scientific, Waltham, MA, USA). Conditions for PCR amplification were as follows: 94°C x 3 min, 35 cycles of 94°C x 30 s, 50°C x 1 min, 72°C x 1 min, 1 cycle of 72°C of 5 min. To amplify DNA fragments containing parts of 5S the primer pair 5’ CAA TTT ATT TGC GCT TCT CTG A 3’ and 5’ ATC CGG TGC ATT AGT GCT G 3’ were used. The forward primer is binding at the noncoding region whereas the reverse primer is binding at the coding region. The obtained PCR product was cloned into the pJet1.2 vector and then labeled by nick translation with Atto488 according to the manufacture instructions (Jena Bioscience, Jena, Germany). DNA fragments containing parts of 45S rDNA were amplified with the primer pair 5’ GAA GTT TGA GGC AAT AAC AGG TCT 3’ and 5’ACC AGC TAC TAG ACG GTT CGA TTA 3’ at 50°C. Both primers are binding at the coding region. The PCR amplicon was also cloned into the pJet1.2 vector and afterwards labeled with Atto550 NT labeling kit (Jena Bioscience, Jena, Germany). The carrot’s transcriptome sequence was used to develop the primers [28].

The slides from the chromosome preparation were washed twice in saline sodium citrate (SSC) and had been processed with 45% acetic acid for 10 min. For post-fixation, the slides had been incubated in 4% formaldehyde for 10 min, washed three times in 2x SSC for 5 min, followed by dehydration in a graded ethanol series 70%, 85% and 96% for 2 min each and air dried afterwards. The hybridization mixture contained 50% deionized formamide, 20% dextran sulfate, 2x SSC and 10 μg/ml salmon sperm DNA. 1 μl of each labeled probe was added to 18 μl hybridization mixture and then had been denatured at 95°C for 10 min, dropped onto slides, covered with cover slips and sealed with rubber cement (Marabu, Ludwigsburg, Germany). Slides with probes had been denatured at 80°C for 2 min and incubated at 37°C in a humid chamber overnight. After hybridization and removing the cover slips, slides had been washed in 2x SSC for 20 min at 58°C, dehydrated in an ethanol concentration (70, 85, 96%); the slides were air dried and counterstained with 12 μl 4′,6′-diamidino-2-phenylindole (DAPI) in Vectashield^®^ (Vector Laboratories, Burlingame, CA, USA). Images were obtained on a microscope Axioimager Z1 (Zeiss, Oberkochen, Germany), capturing each fluorescence dye separately with a cooled CCD camera system Axiocam (Zeiss, Oberkochen, Germany). Pictures were processed and merged by Isis software (Metasystems, Altlussheim, Germany). To determinate the pattern of signals obtained with the 5S rDNA and 45S rDNA probes, five metaphase cells from at least two different root tips were evaluated.

### Flow cytometry detection of relative DNA content

Fresh phylloclade tissue was excised and preserved over night at 4°C in a moist Petri dish. Approximately 0.5 cm^2^ plant material was chopped with a razor blade in 1 ml ice-cold nucleic extraction buffer [29] supplemented with 50 μg DNase-free RNase and 50 μg propidium iodide. *Solanum lycopersicon* ‘Stupické’ (2C = 1.96 pg) [30], and *Pisum sativum* ‘Ctirad’ (2C = 9.09 pg) [31] were used as internal reference standard. The nuclei suspension was poured through a 35 μm Cell-Strainer Cap (Falcon^®^) and was measured using the flow cytometer BD FACS-Calibur^TM^ (Beckton, Dickinson and Company, USA). For each sample 5000–7000 events were registered. At least three individual plants per accession were measured. For seven accessions only one individual plant was available. In these cases, a minimum of three different samples of phylloclade materials were examined. The calculation of the absolute nuclear DNA content based on the formula:

$$Sample\;2C\;DNA\;(pg)=Standard\;2C\;DNA\;amount\;(pg)*\frac{Sample\;G1\;peak\;mean}{Standard\;G1\;peak\;mean}.$$

Statistical analysis was performed using R [32]. Mean values, standard deviation (SD) and coefficient of variation (CV) for the 2C DNA content were calculated for each accession. The genome size was determined considering 1 pg DNA equal to 0.978 x 10^9^ bp [33]. The monoploid genome size (1Cx) was calculated dividing the 2C value by ploidy level [34]. A one-way analysis of variance (ANOVA) and Tukey´s honesty significant difference (HSD) test at P = 0.05 were performed using the 1Cx-values. Correlation’s coefficients were calculated using Excel application.

## Results

### Different ploidy level

**Fig 1:**
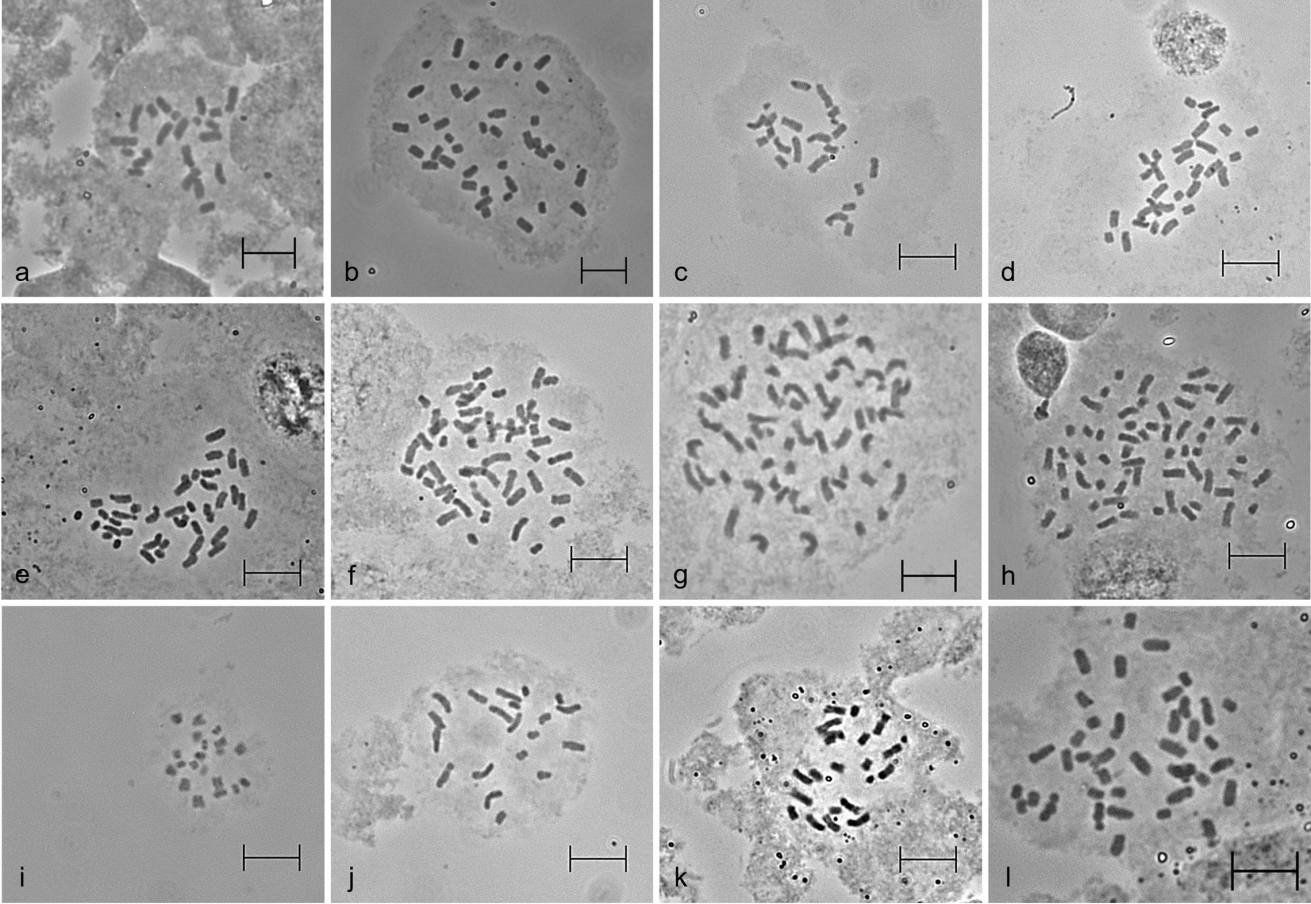
Mitotic metaphase chromosomes in root tip cells. a) *A*. *officinalis* ‘Darlise’ (2n = 2x = 20); b) *A*. *officinalis* ‘Steiners Violetta’ (2n = 4x = 40); c) *A*. *albus* (2n = 2x = 20); d) *A*. *albus* (2n = 3x = 30); e) *A*. *prostratus* 4 (2n = 4x = 40); f) *A*. *amarus* (2n = 5x = 50); g) *A*. *amarus* (2n = 6x = 60); h) *A*. *maritimus* 4 (2n = 6x = 60); i) *A*. *scoparius* (2n = 2x = 20); j) *A*. *stipularis* 3 (2n = 2x = 20); k) *A*. *verticillatus* 2 (2n = 2x = 20); l) *A*. *verticillatus* 1 (2n = 4x = 40); Scale bar = 10 μm.

The karyotype analysis of *Asparagus* species revealed the presence of different chromosome numbers, shown in Figs 1 and S1. All species have the same basic haploid chromosome number x = 10 but they show a variation in the ploidy status. Fifteen diploid (2n = 2x = 20), eight tetraploid (2n = 4x = 40) and nine hexaploid (2n = 6x = 60) were found among the thirty two wild accessions (Table 1). For *A*. *maritimus*, *A*. *prostratus* and *A*. *stipularis*, each four accessions with different geographic origin were examined and all of them show the same ploidy level within the species.

In contrast, different ploidy levels were found in *A*. *densiflorus* and *A*. *verticillatus*: Two accessions of *A*. *densiflorus* were hexaploid, whereas one accession was tetraploid. Additionally, two accessions of *A*. *verticillatus* were diploid but one accession was tetraploid. Furthermore, different numbers of chromosomes were found within one accession. The examination of several plants of *A*. *amarus* showed that some plants were hexaploid while one plant was pentaploid. Similar results were achieved in the species *A*. *albus* L.: two diploid and one triploid plant within the accession.

### Chromosome size variance

During the investigation of the chromosome numbers, a variation of the chromosome size was noticed (Table 2 and S1 Table). The measurement of the chromosomes of the diploid accessions revealed a variation in the chromosome size. The longest chromosome was found in *A*. *officinalis* ‘Darlise’ (5.43 μm) and the shortest one in *A*. *plocamoides* Webb ex Svent. (1.21 μm). Total chromosome length varied in diploid *Asparagus* accessions from 39.6 μm (*A*. *scoparius*) to 69.1 μm (*A*. *verticillatus* 3).

**Table 2:** Determined karyotypic formula of diploid *Asparagus* accessions such as minimum and maximum size of the measured chromosomes.

Species	Karyotypic formula^1^	Chromosome size (μm)		Total Chromosome
		Min. ± SD	Max. ± SD	length (μm)
*A*. *officinalis* ‘Darlise’	4L + 2M + 4S (16)	1.9 ± 0.29	5.4 ± 0.58	68.2 ± 5.62
*A*. *acutifolius*	3L + 3M + 4S (14)	1.9 ± 0.35	4.9 ± 0.75	65.9 ± 10.71
*A*. *africanus*	0L + 3M + 7S (18)	1.4 ± 0.19	3.8 ± 0.74	49.5 ± 5.27
*A*. *albus*	3L + 3M + 4S (19)	1.9 ± 0.35	4.7 ± 0.84	63.9 ± 8.67
*A*. *arborescens*	0L + 4M + 6S (17)	1.5 ± 0.21	3.9 ± 0.58	53.2 ± 7.00
*A*. *plocamoides*	0L + 1M + 9S (32)	1.2 ± 0.15	3.3 ± 0.54	44.5 ± 5.33
*A*. *plumosus*	0L + 4M + 6S (16)	1.4 ± 0.18	3.9 ± 0.48	50.5 ± 5.42
*A*. *ramosissimus*	3L + 2M + 5S (14)	1.7 ± 0.23	5.3 ± 0,56	66.1 ± 4.51
*A*. *scoparius*	0L + 0M + 10S (23)	1.3 ± 0.14	2.8 ± 0.32	39.6 ± 2.54
*A*. *stipularis* 1	1L + 4M + 5S (18)	1.6 ± 0.20	4.3 ± 0.57	57.1 ± 4.97
*A*. *stipularis* 2	2L + 3M + 5S (13)	1.6 ± 0.22	4.7 ± 0.53	59.4 ± 4.83
*A*. *stipularis* 3	3L + 3M + 4S (18)	1.8 ± 0.21	5.1 ± 0.56	67.6 ± 7.35
*A*. *verticillatus* 2	4L + 2M + 4S (19)	2.0 ± 0.23	4.7 ± 0.63	67.3 ± 7.44
*A*. *verticillatus* 3	4L + 2M + 4S (14)	2.0 ± 0.28	5.0 ± 1.00	69.1 ± 11.62
*A*. *sp*.	0L + 0M + 10S (10)	1.2 ± 0.09	2.9 ± 0.43	40.2 ± 4.21

^1^ Classification into three groups according to the chromosome size; small (S) chromosome pair less than 3μm, medium-sized (M) chromosome pair between 3 μm and 4 μm and large (L) chromosome pairs greater than 4 μm; (No of measured metaphase plates).

The chromosomes were classified into three groups based on their size. For *A*. *officinalis* ‘Darlise’ the karyotypic formula 4L + 2M + 4S was estimated. Only the diploid *A*. *verticillatus* accessions show the same karyotypic formula, whereas all other species have a decrease in the number of large chromosomes. The species *A*. *scoparius* and *A*. *sp*. consist only of small chromosomes (0L + 0M + 10S).

### Physical mapping of 45S and 5S rDNA loci

**Fig 2:**
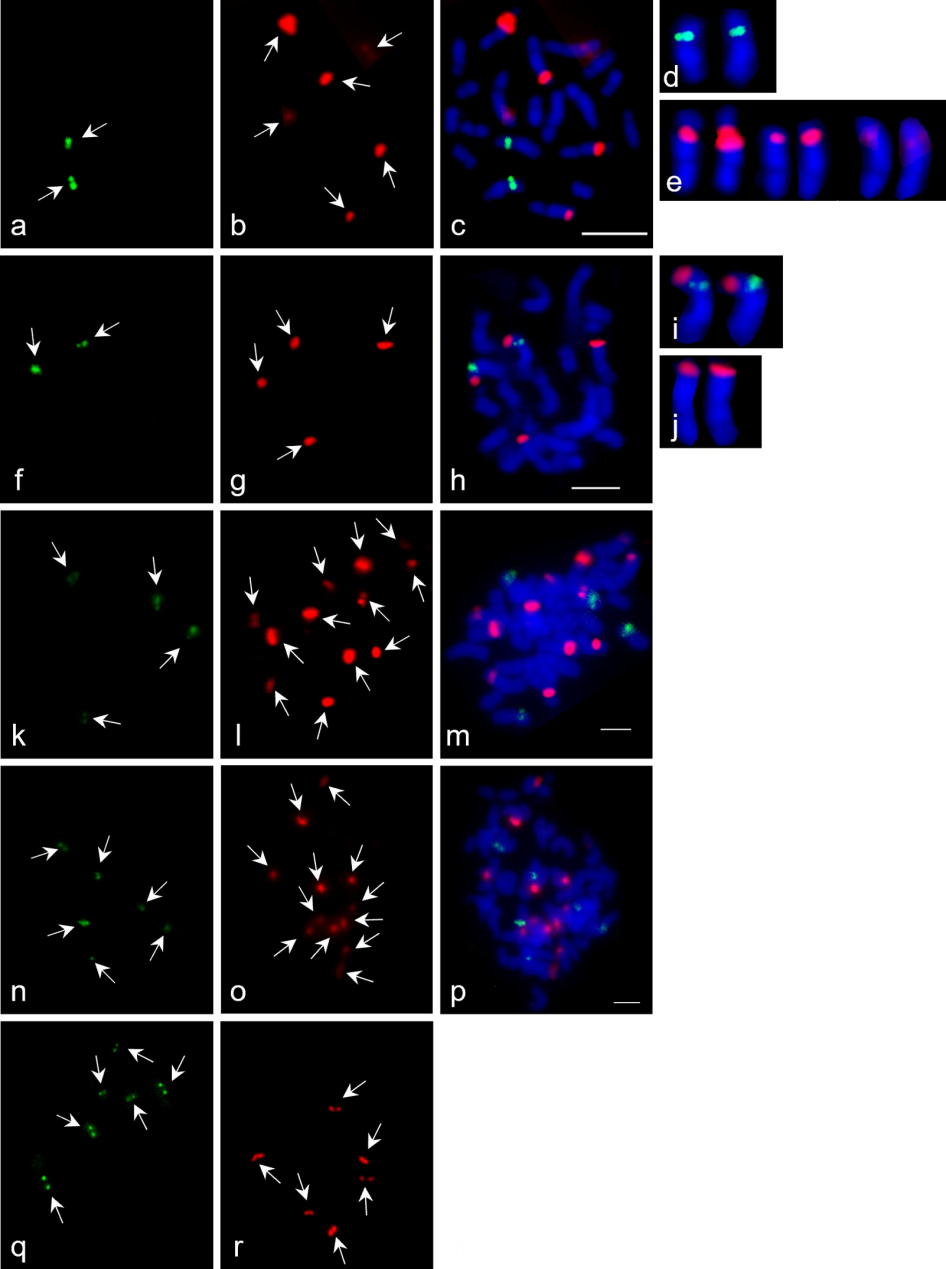
FISH on mitotic metaphase spreads of *Asparagus* species using 5S rDNA (green) and 45S rDNA (red) as probes. The chromosomes were counterstained with DAPI (blue). (a-e) Diploid *A*. *officinalis* ‘Ravel’ showing two signals with 5S rDNA (a, d) and six signals with 45S rDNA (b, e); (c) double FISH with both 5S and 45S rDNA. (f-j) Diploid *A*. *verticillatus* 2 (f) with two 5S rDNA signals (g) and four 45S rDNA signals (h); double FISH with both 5S and 45S rDNA signals, i) one chromosome pair with 5S and 45S rDNA signals, j) one chromosome pair with 45S rDNA. (k-m) Tetraploid *A*. *prostratus* 2 showing k) four 5S rDNA signals, l) twelve 45S rDNA signals and m) double FISH with both 5S and 45S rDNA signals. (n-p) Hexaploid *A*. *maritimus* 4 with n) six 5S rDNA signals, o) twelve 45S rDNA signals and p) double FISH with both 5S and 45S rDNA signals. (q-s) Hexaploid *A*. *aethiopicus* showing six 5S rDNA signals (q), six 45S rDNA signals (r) and double FISH with both 5S and 45S rDNA signals (s). Scale bar = 10 μm.

FISH on mitotic metaphase chromosome plates of 32 different wild *Asparagus* accessions and three *A*. *officinalis* cultivars using 5S rDNA and 45S rDNA as probes are shown in Figs 2 and S2–S6). The results are summarized in Tables 3 and 4. The physical mapping of 5S and 45S rDNA genes reveals a high variability.

**Table 3:** *Asparagus* accessions with their calculated 1Cx DNA content estimated with flow cytometry using *S*. *lycopersicon* as internal standard and their number of 5S and 45S rDNA loci.

Accession	Ploidy	1Cx DNA content^1^ Mean (pg) ± SD	Tukey Grouping^2^	5S rDNA^3^	45S rDNA^3^
*A*. *acutifolius*	2x	1.35 ± 0.05	EFG	2	4
*A*. *aethiopicus*	6x	0.86 ± 0.04	NO	6	6
*A*. *albus*	2x	1.23 ± 0.05	IJK	2	2
*A*. *amarus*	6x	1.37 ± 0.03	EF	6	12
*A*. *arborescens*	2x	1.32 ± 0.02	EFGH	2	4
*A*. *densiflorus* 1	6x	0.84 ± 0.02	NO	6	6
*A*. *densiflorus* 2	6x	0.85 ± 0.02	NO	6	6
*A*. *densiflorus* 3	4x	0.89 ± 0.03	N	4	4
*A*. *maritimus* 1	6x	1.33 ± 0.01	EFGH	6	12
*A*. *maritimus* 2	6x	1.30 ± 0.02	FGHI	6	12
*A*. *maritimus* 3	6x	1.28 ± 0.04	HIJ	6	12
*A*. *maritimus* 4	6x	1.29 ± 0.02	GHIJ	6	12
*A*. *officinalis* ‘Darlise’	2x	1.47 ± 0.04	D	2	6
*A*. *officinalis* ‘Ravel’	2x	1.53 ± 0.01	BC	2	6
*A*. *officinalis* ‘Steiners Violetta’	4x	1.59 ± 0.01	AB	4	12
*A*. *pastorianus*	4x	1.40 ± 0.01	E	4	8
*A*. *plocamoides*	2x	0.71 ± 0.02	Q	2	8
*A*. *prostratus* 1	4x	1.48 ± 0.02	CD	4	12
*A*. *prostratus* 2	4x	1.53 ± 0.01	BCD	4	12
*A*. *prostratus* 3	4x	1.51 ± 0.01	BCD	4	8
*A*. *prostratus* 4	4x	1.54 ± 0.02	BC	4	8
*A*. *pseudoscaber*	6x	1.28 ± 0.01	GHIJ	6	12
*A*. *ramosissimus*	2x	1.15 ± 0.003	LM	2	4
*A*. *scoparius*	2x	0.73 ± 0.003	PQ	2	6
*A*. *setaceus*	4x	0.74 ± 0.01	PQ	4	16
*A*. *stipularis* 1	2x	0.81 ± 0.01	OP	2	2
*A*. *stipularis* 2	2x	1.13 ± 0.01	M	2	2
*A*. *stipularis* 3	2x	1.21 ± 0.005	JKL	2	2
*A*. *stipularis* 4	2x	1.19 ± 0.04	KLM	2	2
*A*. *verticillatus* 1	4x	1.52 ± 0.002	BCD	4	12
*A*. *verticillatus* 2	2x	1.65 ± 0.01	A	2	4
*A*. *verticillatus* 3	2x	1.54 ± 0.01	BC	2	4

^**1**^ Monoploid DNA content was calculated 2C DNA content / ploidy level;

^**2**^ Tukey results, means with the same letters did not differ significantly (P ≤ 0.05);

^**3**^ Number of FISH signals in a mitotic metaphase plate (2n).

**Table 4:** *Asparagus* accessions with their calculated 1Cx DNA content estimated with flow cytometry using *P*. *sativum* as internal standard and their number of 5S and 45S rDNA loci.

Accession	Ploidy	1Cx DNA content^1^ Mean (pg) ± SD	Tukey Grouping^2^	5S rDNA^3^	45S rDNA^3^
*A*. *africanus*	2x	1.05 ± 0.01	B	2	4
*A*. *officinalis* ‘Darlise’	2x	1.52 ± 0.02	A	2	6
*A*. *plumosus*	2x	1.06 ± 0.01	B	2	2
*A*. *sp*.	2x	0.93 ± 0.02	C	2	4

^**1**^ Monoploid DNA content was calculated 2C DNA content / ploidy level;

^**2**^ Tukey results, means with the same letters did not differ significantly (P ≤ 0.05);

^**3**^ Number of FISH signals in a mitotic metaphase plate (2n).

A homogenous pattern of signals obtained with the 5S rDNA probe was observed in all accessions. In diploid accessions two 5S rDNA signals were detected. Furthermore, four signals of 5S rDNA were discovered in all tetraploid accessions and six signals in all hexaploid species.

The analysis of pattern of signals obtained with the 45S rDNA probe revealed a variation in the number and the positions. In diploid accessions, the number of 45S rDNA signals ranges from two to eight. Two 45S rDNA signals were noticed in *A*. *albus*, *A*. *plumosus* and all accessions of *A*. *stipularis*. However, in *A*. *acutifolius*, *A*. *africanus* Lam., *A*. *arborescens* Willd. Ex Schult. & Schult.f., *A*. *ramosissimus* and *A*. *sp*., four signals were observed while six signals were detected in both diploid *A*. *officinalis* cultivars (Fig 2A–2c) and in the diploid *A*. *verticillatus* accession (Fig 2D–2H). Moreover, eight loci of 45S rDNA were found in *A*. *plocamoides* and *A*. *scoparius*. In tetraploid *Asparagus* accessions four to sixteen 45S rDNA signals have been observed. Tetraploid *A*. *densiflorus* has four 45S sites whereas *A*. *pastorianus* Webb & Berthel. has eight 45S loci. The tetraploid *A*. *prostratus* accessions show a variation in the number of 45S signals. In each of two accessions, eight and twelve 45S loci were detected (Fig 2I–2K). In the tetraploid *A*. *officinalis* ‘Steiners Violetta’ and the tetraploid *A*. *verticillatus* accession, twelve 45S rDNA signals have been observed. Sixteen 45S sites were revealed in tetraploid *A*. *setaceus* (Kunth) Jessop. The analysis of hexaploid accessions showed either six or twelve 45S rDNA sites. Moreover, six signals of 45S were found in hexaploid *A*. *aethiopicus* L. (Fig 2O–2Q) and *A*. *densiflorus* whereas all accessions of *A*. *maritimus* (Fig 2L–2N) and *A*. *pseudoscaber* showed twelve 45S rDNA sites.

In almost all accessions, the 5S and 45S rDNA signals are localized on different chromosomes. Yet, there were a few exceptions: In *A*. *africanus*, *A*. *plocamoides*, *A*. *sp*. and in both diploid *A*. *verticillatus* (Fig 2D–2H) accessions a 5S and one 45S rDNA locus occurred on the same chromosome.

### Determination of nuclear DNA content

The DNA content of the analyzed *Asparagus* species ranges from 1.43 pg (*A*. *plocamoides*, diploid) to 8.24 pg (*A*. *amarus*, hexaploid) DNA per nucleus. Two different internal standards were used to determine the genome size of three *A*. *officinalis* cultivars and 32 wild *Asparagus* accessions due to the very dissimilar genomic sizes. An overview of genome sizes in all accessions investigated is given in Table 1. For seven accessions only one plant was available, so three examinations of the same plant were analyzed on different days. The results varied at least as much as the samples taken from different plants.

The coefficient of variation varies from 0.16% (*A*. *verticillatus* 1, tetraploid) to 4.51% (*A*. *aethiopicus*, hexaploid). The nuclear DNA content of *A*. *officinalis* ‘Darlise’ was 2.95 pg using *S*. *lycopersicon* ‘Stupické’ (2C = 1.96 pg) as the internal standard. When *P*. *sativum* ‘Ctirad’ (2C = 9.09 pg) was used as the internal standard the genome size of *A*. *officinalis* ‘Darlise’ was 3.04 pg. The variability of the different 2C DNA values of *A*. *officinalis* ‘Darlise’ was tested using one-way ANOVA. The results showed that 2C-values calculated with different internal standards had significant differences among each other (p ≤ 0.01). Therefore, the statistical analysis has been divided based on the internal standards used.

The calculated 1Cx–values varied from 0.71 pg (*A*. *plocamoides*) to 1.65 pg (*A*. *verticillatus* 2) (Tables 3 and 4). The ANOVA analysis showed significant differences for 1Cx-values (F = 558.9, P = <2e-16) between the species. The Tukey grouping revealed 17 different groups.

In the case of five wild *Asparagus* species more than one accession belonging to the same species were analyzed, which allows the interpretation of intraspecific variation. The 1Cx-value of *A*. *densiflorus*, *A*. *maritimus* and *A*. *prostratus* showed no significant difference in the DNA content within the tested accessions. Intraspecific variation was noticed in the species *A*. *verticillatus* and *A*. *stipularis* (P<0.05). The 1Cx value for all three *A*. *verticillatus* accessions showed an 8% difference ranging from 1.52 pg/1Cx to 1.65 pg/1Cx. The accessions from *A*. *stipularis* varied from 0.81 pg/1Cx to 1.21 pg/1Cx, which signifies a variation of 33%. A variation of 7.55% were measured between the three tested *A*. *officinalis* cultivars.

The relationship between total chromosome length of diploid *Asparagus* accessions and their genome size was high correlated (r = 0.83, p = 0.01) (S1 Table).

## Discussion

Different ploidy levels have already been described for several *Asparagus* species. The diploidy (2n = 2x = 20) of *A*. *acutifolius*, *A*. *africanus*, *A*. *albus*, *A*. *arborescens*, *A*. *plocamoides*, *A*. *plumosus*, *A*. *scoparius*, *A*. *stipularis* and *A*. *verticillatus* as well as the tetraploid level (2n = 4x = 40) of *A*. *pastorianus* is published in Index to Plant Chromosome Numbers (IPCN; http://www.tropicos.org/Project/IPCN) and could be verified in our material. For *A*. *acutifolius*, a diploid set of chromosomes (2n = 2x = 20) has been identified, which corresponds to IPCN, whereas in several other publications a tetraploid ploidy level was described [3, 21, 25, 35]. For *A*. *densiflorus* IPCN indicates a tetraploid state, in this study one tetraploid and two hexaploid (2n = 6x = 60) accessions were found. The detected hexaploidy for *A*. *amarus*, *A*. *maritimus* and *A*. *pseudoscaber* agrees with the published data [3, 21, 36]. A tetraploid level was approved for the four *A*. *prostratus* accessions, confirming the study of Tutin *et al*. [35], Kay *et al*. [24] and Castro *et al*. [21]. For *A*. *setaceus* a diploid ploidy level is described [37, 38], whereas our research identified a tetraploid accession. No information of ploidy has been published for the species *A*. *ramosissimus* Baker and *A*. *aethiopicus*, for which we identified a diploid (2n = 2x = 20) and a hexaploid accession (2n = 6x = 60), respectively.

Plants with different ploidy within the same population are very common in the *Asparagus* genus. For the Spanish landrace ‘Morado de Huetor’ triploid, pentaploid, hexaploid and octoploid plants have been found [39]. Ozaki *et al*. [40] discovered a spontaneous triploid asparagus plant from crosses with diploid parents. For the accessions *A*. *amarus* and *A*. *albus* different sets of chromosomes have been observed. In general, polyploidization is discussed as an important mechanism in the evolution [41, 42]. So far, no correlation between geographical distribution and ploidy has been found [43]. In a field with diploid garden asparagus several tetraploid plants were selected that emerged from unreduced gametes [18]. In addition to the two diploid cultivars, we present the tetraploid *A*. *officinalis* ‘Steiners Violetta’. The high plasticity of the *Asparagus* genome, even inside species, underscores the need of a precise characterization of each plant at the beginning of the crossing programs.

We propose the karyotype formula of *A*. *officinalis* 4L+ 2M+ 4S dividing the chromosomes according to their size. Chromosomes smaller than 3 μm were classified as small (S). Between 3μm and 4μm large chromosomes were assigned as medium-sized (M) chromosomes. In the large-sized (L) group, the chromosomes were classified larger than 4 μm. Further karyotypic formulas were already reported based on the classification of the chromosomes by Löptien [44] stained with Giemsa. The karyotypic formula published by De Melo and Guerra [45] agrees with the one determined in this work. Löptien [44] and Deng *et al*. [46] identified five large chromosomes (L) in total, only one medium sized chromosome (M) and five small chromosomes (S). The difference in the karyotypic formula can occur due to an intraspecific variation of *A*. *officinalis* cultivars or due to a different measurement method used to classify the chromosomes.

A few differences in chromosome length for *A*. *officinalis* has already been published. The values for *A*. *officinalis* determined in this work were between 1.92 μm and 5.43 μm. These results are in concordance with the data published by Mukhopadhyay and Ray [47], which describe the chromosomal length between 0.92 and 5.83 μm. Furthermore, their defined total chromosome length of 61.76 μm corresponds to ours of 68.2 μm. Akter *et al*. [37] measured only really small chromosomes with a length ranging from 0.48 to 1.58 μm. However, he examined an *A*. *officinalis* accession with 22 chromosomes, suggesting that this plant is closely related to *A*. *officinalis* or a new cytotype. In this study, *A*. *scoparius* Lowe and *A*. *sp*. have only small chromosomes ranging from 1.22 μm and 2.87 μm. Chromosomes smaller than 1 μm were not found. It could again be assumed that the causes of the different results are due to intraspecific differences in the cultivars used or due to the different measurement methods.

Chromosome painting is an additional tool for karyotyping especially when the chromosome shape is difficult to distinguish. Hybridization with probes of fluorescently labeled 5S and 45S ribosomal RNA genes was used, which are well-known regarding their number of repetitive copies. The in- situ localization of 5S and 45S rDNA in the chromosome complement has already been shown for a wide range of plants [48]. Moreno *et al*. [49] used FISH to analyze the localization of 5S and 45S rDNA loci on flow-sorted chromosomes of *A*. *officinalis* and revealed that the sex determination locus is associated with 5S rDNA locus. In previous studies with *A*. *officinalis*, Reamon-Büttner *et al*. [50] detected 5S rDNA signals on a single chromosome and with 45S rDNA probes they found six sites of hybridization on three pairs of chromosomes. De Melo and Guerra [45] confirmed six sites of 45S rDNA on large chromosomes in four *A*. *officinalis* cultivars. The reports of Deng *et al*. [46, 51] show the technical difficulties of FISH for *Asparagus*. Besides the small chromosomes, the accessibility of the probe to the chromosomal target DNA can be limited due to the folding or position inside the chromosome. Firstly, the authors claimed that they found six to eight 5S rDNA sites on four pairs of chromosomes and six 45S rDNA signals. Two years later Deng *et al*. [51] corrected themselves to have found six signals of 45 rDNA and two signals 5S rDNA which is consistent with other results [43, 49, 50] as well as this study´s outcome. For the *Asparagus* wild species *A*. *prostratus* (4x) and *A*. *maritimus* (6x) two 45S rDNA loci per basic chromosome set (x) were identified [52]. Our work confirms these findings for all *A*. *maritimus* accessions and for two *A*. *prostratus* accessions (3 and 4). But in the other two tetraploid *A*. *protratus* accessions (1 and 2), we observed three 45S rDNA signals. All four *A*. *prostratus* accessions should be analyzed further, because a pre-breeding program for the transmission of AV1 resistance with this material is in progress.

Mousavizadeh *et al*. [43] studied the wild species *A*. *persicus*, *A*. *verticillatus* and *A*. *breslerianus*. For *A*. *verticillatus* they noticed two signals for the 45S rDNA probe and one signal 5S rDNA. The 5S and one 45S rDNA signal were located on the same chromosome. This result was confirmed for both tested diploid *A*. *verticillatus* accessions. Surprisingly, the tetraploid accession revealed twelve sites of 45S rDNA instead the expected eight 45S rDNA signals after the chromosome duplication. Furthermore, the 5S and 45S rDNA signals were located on different chromosomes. Additional investigations are necessary to analyze the tetraploid *A*. *verticillatus* accession. In this study, the localization of the 5S rDNA signal with one 45S rDNA signal on the same chromosome was not only found for *A*. *verticillatus* but also in *A*. *sp*., *A*. *africanus*, and *A*. *plocamoides*, respectively. The occurrence of both sites on the same chromosome has been reported in several species. In general, it can be said that it occurred more often in karyotypes with multiple sites [48].

The question of whether polyploid *Asparagus* accessions could have evolved from diploid species (autopolyploid) or by hybridization of different species (allopolyploid) has not yet been clarified. In this study the number of signals of 5S and 45S rDNA signals followed the ploidy. That could be a clue that all polyploid accessions derived from whole genome polyploidization and therefore are autopolyploids. Only the FISH results for the tetraploid *A*. *verticillatus* needs more clarification concerning a putative allopolyploid origin. So Mousavizadeh *et al*. [43] assumed an allopolyploid octoploid *A*. *breslerianus* because they found two out of the four pairs of 5S rDNA sites adjacent to 45S rDNA sites.

In the present work, the 2C DNA content was defined for three *A*. *officinalis* cultivars and 32 wild asparagus accessions of various species. The large variability of genome size inside the genus *Asparagus* is caused by both the polyploidization and the variation of the chromosome size. Due to the very different DNA content across the species the use of an *Asparagus* plant of the same species with a given chromosome number estimated by chromosome counting as standard remains a mandatory requirement for the ploidy estimation in *Asparagus* species by flow cytometry. Different ploidies within the same species were in our study morphologically indistinguishable, but sound knowledge about is very essential for breeding efforts. Furthermore, for the determination of the absolute DNA content known internal standards such as pea or tomato are suitable.

The measured 2C DNA content of *A*. *officinalis* cultivars ranges from 2.95 to 3.06 pg. It fits to previously published genome sizes of *A*. *officinalis*. Arumuganathan and Earle [23] estimated a 2C DNA content of 2.71 pg, Štajner *et al*. [3] estimated 2.78 pg for an *A*. *officinalis* cultivar and 2.92 pg for a wild *A*. *officinalis*, whereas Bennett and Leitch [53] announced 3.6 pg. The differences could occur due technical paricularities of the laboratories such as the use of different internal standards, differences in the protocol (e.g. different extraction buffer), different flow- cytometers and so on [30, 31, 54]. There exists also undoubtedly a genotypic variation of asparagus accessions. Additionally, deviations in nuclear DNA content could indicate aneuploidy [55].

The relationship between chromosome size and DNA content determined in this work has already been described, for example in the genus *Knautia* [56]. In the case of *Asparagus*, a high correlation has so far been found between the ploidy level of *A*. *officinalis* cultivars and their genome size [40]. Such as a connection between total chromosome length and total chromosome volume was described [47].

Very few genome size determinations of wild *Asparagus* accessions have been published to date. The haploid genome sizes of *A*. *verticillatus* (1.52 pg), *A*. *acutifolius* (1.43 pg), *A*. *maritimus* (1.28, 1.31 pg) and *A*. *densiflorus* (0.85 pg) published by Štajner et al. [3] are very close to the results of this study. Štajner *et al*. [3] indicate that European *Asparagus* species have a larger 1Cx value compared to southern African *Asparagus* species due to high genetic dissimilarities. Further African *Asparagus* species are being presented in this work. The results show that among the African species are those with very low 1Cx DNA content such as *A*. *plocamoides* with only 0.71 pg, and those with a DNA content up to 1.40 pg (e.g. *A*. *pastorianus*, 1.40 pg). In die European clade the 1 Cx values varies from 1.23 (*A*. *albus*) to 1.65 pg (*A*. *verticillatus* 2). The DNA content of European accessions is in most cases higher, however there are also *Asparagus* accessions with a similar 1Cx value in both groups. For example, the 1 Cx value of *A*. *arborescens* (1.32 pg) is comparable with *A*. *maritimus* 1 (1.33 pg).

*A*. *officinalis* and *A*. *verticillatus* harbor the most large chromosomes and at the same time belong to the species with the highest 1Cx DNA content. In *A*. *scoparius* only small chromosomes were found which corresponds with its low 1Cx DNA content of 0.73 pg. For *A*. *plumosus* Štajner *et al*. [3] noticed 0.72 pg (1Cx) whereas this study found 1.06 pg. The significant difference could be due to the different origin of both accessions. Our plant material originated from Cuba (invasive weed), whereas Štajner and coworker got this species as two ornamental cultivars from Anderson’s SeedCo (California) or from Walz Samen GmbH, a seed provider in Germany. *A*. *plumosus* has also been listed as a synonym of *A*. *setaceus* in several publications [38, 57].

Four accessions of *A*. *stipularis* were investigated and it is remarkable that one accession (*A*. *stipularis* 1) differs significantly from the three others. On the one hand, the DNA content of *A*. *stipularis* 1 with 1.62 pg (2Cx) versus 2.26–2.42 pg (*A*. *stipularis* 2–4) is much lower. Furthermore the karyotypic formula of *A*. *stipularis* varies. *A*. *stipularis* 1 has chromosomes with the size 1L + 4M + 5S, whereas *A*. *stipularis* 3 contains 3L + 3M + 4S chromosomes. Also the total chromosome length of *A*. *stipularis* 1 is with 57.1 μm noticeable shorter than that of *A*. *stipularis* 3 with 67.6 μm. The results of this study indicates that there is a wide intraspecific variation in *A*. *stipularis*. Morphologically the plants are indistinguishable. Already by molecular investigation with SSR markers, a significant genetic diversity among the *A*. *stipularis* accessions has been found [13].

The high variability of the karyotypes and the very different 1Cx DNA content of the *Asparagus* species connected with a consistent basic chromosome number of ten are notheworthy and needs more clarification. Abundant transposable elements (TE) could contribute to the large differences in genome size observed between *Asparagus* species [3, 58]. Retrotransposon proliferation is the reason that the genome size of dioecious *Asparagus* species is in most cases larger than in hermaphroditic species [59]. Suzuki *et al*. [60] used random BAC FISH clones of asparagus to elucidate the distribution of repetitive DNA elements in *A*. *officinalis*. Mainly dispersed types of signals were detected indicating only a small proportion of repetitive sequences in the *A*. *officinalis* genome. The data of Li *et*. *al* [38] show that 69% of the *A*. *officinalis* genome is occupied by repetitive sequences, whereas the wild relative *A*. *setaceus* consists of 65.59% of repetitive sequences. The examination of three *Asparagus* species (*A*. *officinalis*, *A*. *racemosus* and *A*. *setaceus*) also shows different structural abnormalties such as deletion, tandem duplications and dispersed distribution of GC- and AT-rich repetitive sequences [37].

## Conclusion

The assessment of 32 wild *Asparagus* accessions and three *A*. *officinalis* cultivars by classical chromosome counting, FISH and flow cytometry shows the enormous plasticity of the genus. Due to minor or no morphological differences between some accessions it is worth to analyze the accessions thoroughly, especially if they should serve as genetic resource for breeding efforts.

## Supporting information

S1 Table Origin data of measuring the chromosome size and calculated correlation between total chromosome length of diploid *Asparagus* accessions and their genome size. (XLSX)

S1 Fig Mitotic metaphase chromosomes in root tip cells. a) *A*. *acutifilius* (2n = 2x = 20); b) *A*. *aethiopicus* (2n = 6x = 60); c) *A*. *africanus* (2n = 2x = 20); d) *A*. *arborescens* (2n = 2x = 20); e) *A*. *densiflorus* 1 (2n = 6x = 60); t) *A*. *densiflorus* 2 (2n = 6x = 60); g) *A*. *densiflorus* 3 (2n = 4x = 40); h) *A*. *maritimus* 1 (2n = 6x = 60); i) *A*. *maritimus* 2 (2n = 6x = 60); j) *A*. *maritimus* 3 (2n = 6x = 60); k) *A*. *officinalis* ‘Ravel’ (2n = 2x = 20); l) *A*. *pastorianus* (2n = 4x = 40); m) *A*. *plocamoides* (2n = 2x = 20), n) *A*. *plumosus* (2n = 2x = 20); o) *A*. *prostratus* 1 (2n = 4x = 40), p) *A*. *prostratus* 2 (2n = 4x = 40); q) *A*. *prostratus* 3 (2n = 4x = 40); r) *A*. *pseudoscaber* (2n = 6x = 60); s) *A*. *ramossimus* (2n = 2x = 20); t) *A*. *setaceus* (2n = 4x = 40); u) *A*. *stipluaris* 1 (2n = 2x = 20); *v) A*. *stipularis* 2 (2n = 2x = 20); w) *A*. *stipularis* 4 (2n = 2x = 20); x) *A*. *verticillatus* 3 (2n = 2x = 20); y) *A*. *sp*. (2n = 2x = 20); Scale bar = 10 μm.
(TIF)

S2 Fig FISH on mitotic metaphase spreads of *Asparagus* species using 5S rDNA (green) and 45S rDNA (red) as probes. The chromosomes were counterstained with DAPI (blue). (a1—a6) Diploid *A*. *acutifolius* (a3) with two 5S rDNA signals (a1; a4) and four 45S rDNA signals (a2; a5; a6). Two 5S rDNA signals (b1; b4) and four 45S rDNA (b2; b5) were found in diploid *A*. *africanus* (b3). Diploid *A*. *albus* (c3) with two 5S rDNA signals (c1; c4) and two 45S rDNA signals (C2; c5). Six 5S rDNA (d1) and 12 45S rDNA (d2) signals were detected in hexaploid *A*. *amarus* (d3). Diploid *A*. *arborescens* (e3) has two 5S rDNA (e1; e4) and four 45S rDNA (e2-, e5; e6) signals. Six 5S rDNA (f1) and six 45S rDNA (f2) signals were found in hexaploid *A*. *densiflorus* 1. Scale bar = 10 μm.
(PDF)

S3 Fig FISH on mitotic metaphase spreads of *Asparagus* species using 5S rDNA (green) and 45S rDNA (red) as probes. The chromosomes were counterstained with DAPI (blue). Hexaploid *A*. *densiflorus* 2 (g3) has six 5S rDNA (g1) and six 45S rDNA (g2) signals. For tetraploid *A*. *densiflorus* 3 (h3) four 5S rDNA (h1) and four 45S rDNA (h2) signals were found. Hexaploid *A*. *maritimus* 1–3 (i3; j3; k3) has six 5S rDNA (i1; j1; k1) and twelve 45S rDNA signals (i2; j2; k2). For diploid *A*. *officinalis* ‘Darlise’ (l3) two 5S rDNA (l1; l4) and six 45S rDNA (l2; l5) signals were detected. Scale bar = 10 μm.
(PDF)

S4 Fig FISH on mitotic metaphase spreads of *Asparagus* species using 5S rDNA (green) and 45S rDNA (red) as probes. The chromosomes were counterstained with DAPI (blue). Tetraploid *A*. *officinalis* `Steiners Violetta´ (m3) has four 5S rDNA (m1) and twelve 45S rDNA (m2) signals. Tetraploid *A*. *pastorianus* (n3) harbors four 5S rDNA (n1) and eight 45S rDNA signals. Two 5S rDNA (o1; o4) and eight 45S rDNA (o2; o4; o5) signals were detected for diploid *A*. *plocamoides* (o3). Diploid *A*. *plumosus* (p3) has two 5S rDNA (p1; p4) and two 45 S rDNA (p2; p5) signals. Four 5S rDNA (q1) and twelve 45s rDNA signals were found in tetraploid *A*. *prostratus* 1 (q3). Tetraploid *A*. *prostratus* 3 (r3) with four 5S rDNA (r1) and eight 45S rDNA signals (r2). Scale bar = 10 μm.
(PDF)

S5 Fig FISH on mitotic metaphase spreads of *Asparagus* species using 5S rDNA (green) and 45S rDNA (red) as probes. The chromosomes were counterstained with DAPI (blue). Tetraploid *A*. *prostratus* 4 (a3) harbors four 5S rDNA (a1) and eight 45S rDNA (a2) signals. Six 5S rDNA (b1) and twelve 45S rDNA signals were detected in hexaploid *A*. *pseudoscaber* (b3). Diploid *A*. *ramosissimus* (c3) has two 5S rDNA (c1; c4) and 45S rDNA (c2; c5) signals. Two 5S rDNA (d1; d4) and six 45S rDNA (d2; d5) signals were found in diploid *A*. *scoparius* (d3). Tetraploid *A*. *setaceus* (e3) has four 5S rDNA (e1) and sixteen 45S rDNA (e2) signals. Two 5S rDNA (f1; f4) and two 45S rDNA (f2; f5) signals were detected in diploid *A*. *stipularis* 1 (f3). Scale bar = 10 μm.
(PDF)

S6 Fig FISH on mitotic metaphase spreads of *Asparagus* species using 5S rDNA (green) and 45S rDNA (red) as probes. The chromosomes were counterstained with DAPI (blue). Diploid *A*. *stipularis* 2–4 (g3; h3; i3) has two 5S rDNA (g1; g4; h1; i3; i4) and two 45S rDNA (g2; g5; h2; i2) signals. Four 5S rDNA (j1) and twelve 45S rDNA (j2) signals were detected for tetraploid *A*. *verticillatus* 1 (j3). In diploid *A*. *verticillatus* 3 (k3) two 5S rDNA (k1; k4) and four 45S rDNA (k2; k4; k5) signals were found. Diploid *A*. *sp*. (l3) has two 5S rDNA and four 45S rDNA signals. Scale bar = 10 μm.
(PDF)
