# Supplementary figures and images for: Karyological and nuclear DNA content variation of the genus Asparagus
Source: PLoS One. 2022 Mar 16;17(3):e0265405. doi: 10.1371/journal.pone.0265405 (PMC8926174; doi:10.1371/journal.pone.0265405)

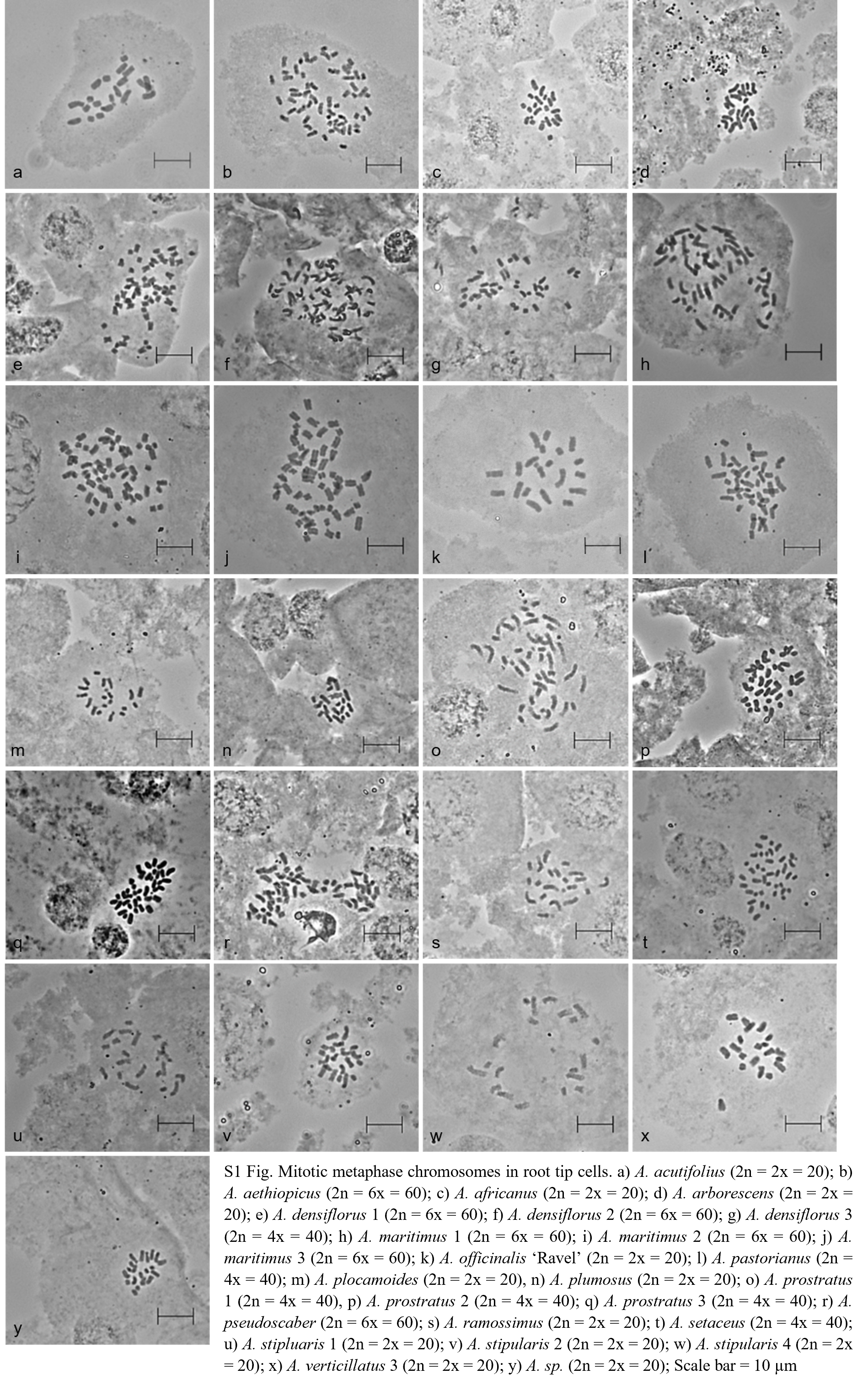

Supplement: S1 Fig — a) A. acutifilius (2n = 2x = 20); b) A. aethiopicus (2n = 6x = 60); c) A. africanus (2n = 2x = 20); d) A. arborescens (2n = 2x = 20); e) A. densiflorus 1 (2n = 6x = 60); t) A. densiflorus 2 (2n = 6x = 60); g) A. densiflorus 3 (2n = 4x = 40); h) A. maritimus 1 (2n = 6x = 60); i) A. maritimus 2 (2n = 6x = 60); j) A. maritimus 3 (2n = 6x = 60); k) A. officinalis ‘Ravel’ (2n = 2x = 20); l) A. pastorianus (2n = 4x = 40); m) A. plocamoides (2n = 2x = 20), n) A. plumosus (2n = 2x = 20); o) A. prostratus 1 (2n = 4x = 40), p) A. prostratus 2 (2n = 4x = 40); q) A. prostratus 3 (2n = 4x = 40); r) A. pseudoscaber (2n = 6x = 60); s) A. ramossimus (2n = 2x = 20); t) A. setaceus (2n = 4x = 40); u) A. stipluaris 1 (2n = 2x = 20); v) A. stipularis 2 (2n = 2x = 20); w) A. stipularis 4 (2n = 2x = 20); x) A. verticillatus 3 (2n = 2x = 20); y) A. sp. (2n = 2x = 20); Scale bar = 10 μm. (TIF) [file pone.0265405.s002.tif]
