# Supplementary material for: Karyological and nuclear DNA content variation of the genus Asparagus
Source: PLoS One. 2022 Mar 16;17(3):e0265405. doi: 10.1371/journal.pone.0265405 (PMC8926174; doi:10.1371/journal.pone.0265405)
Supplement: S2 Fig — The chromosomes were counterstained with DAPI (blue). (a1—a6) Diploid A. acutifolius (a3) with two 5S rDNA signals (a1; a4) and four 45S rDNA signals (a2; a5; a6). Two 5S rDNA signals (b1; b4) and four 45S rDNA (b2; b5) were found in diploid A. africanus (b3). Diploid A. albus (c3) with two 5S rDNA signals (c1; c4) and two 45S rDNA signals (C2; c5). Six 5S rDNA (d1) and 12 45S rDNA (d2) signals were detected in hexaploid A. amarus (d3). Diploid A. arborescens (e3) has two 5S rDNA (e1; e4) and four 45S rDNA (e2-, e5; e6) signals. Six 5S rDNA (f1) and six 45S rDNA (f2) signals were found in hexaploid A. densiflorus 1. Scale bar = 10 μm. (PDF) [file pone.0265405.s003.pdf]

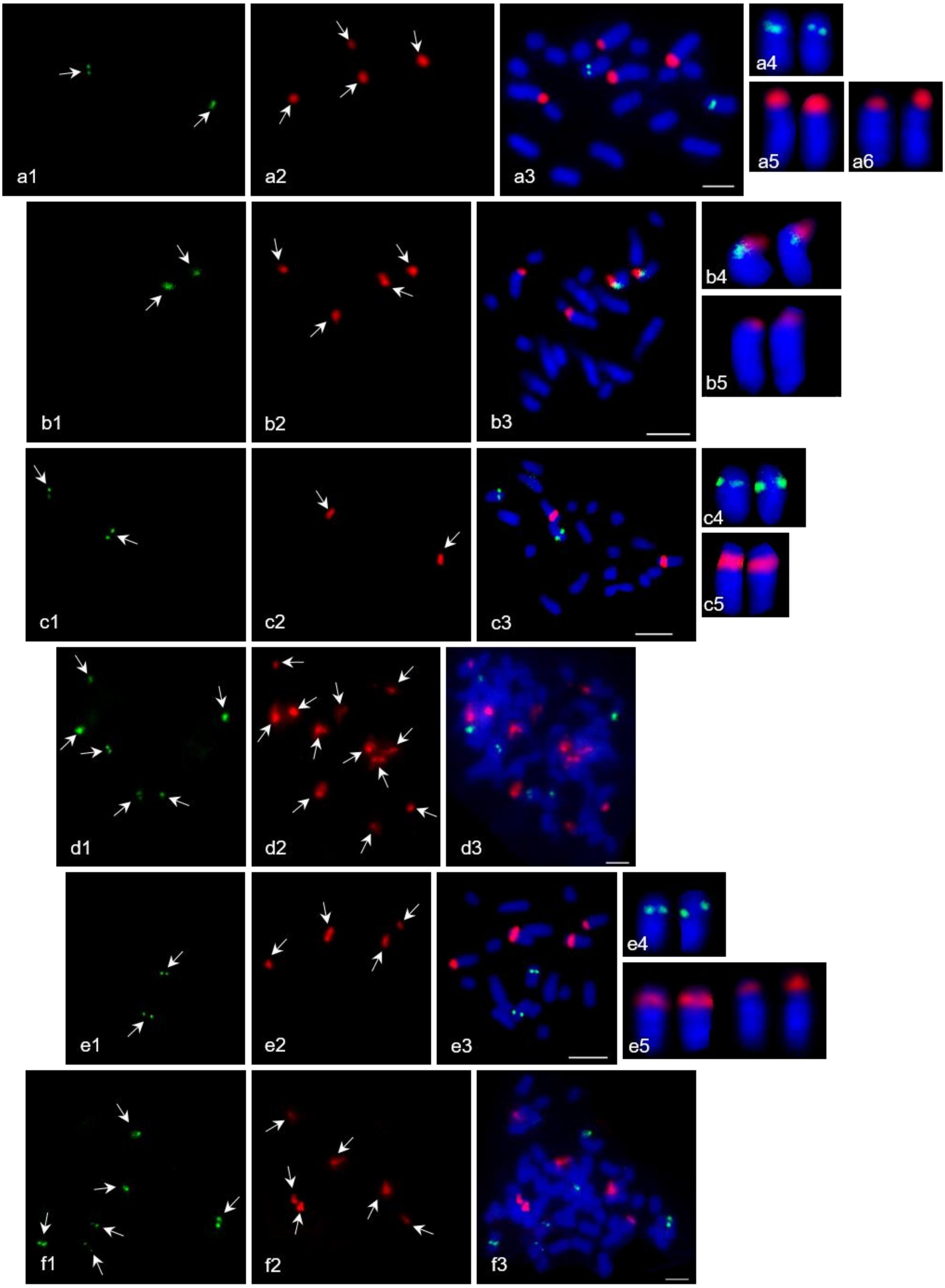

S2 Fig. FISH on mitotic metaphase spreads of *Asparagus* species using 5S rDNA (green) and 45S rDNA (red) as probes. The chromosomes were counterstained with DAPI (blue). (a1 - a6) Diploid *A. acutifolius* (a3) with two 5S rDNA signals (a1; a4) and four 45S rDNA signals (a2; a5; a6). Two 5S rDNA signals (b1; b4) and four 45S rDNA (b2; b5) were found in diploid *A. africanus* (b3). Diploid *A. albus* (c3) with two 5S rDNA signals (c1; c4) and two 45S rDNA signals (C2; c5). Six 5S rDNA (d1) and 12 45S rDNA (d2) signals were detected in hexaploid *A. amarus* (d3). Diploid *A. arborescens* (e3) has two 5S rDNA (e1; e4) and four 45S rDNA (e2-, e5; e6) signals. Six 5S rDNA (f1) and six 45S rDNA (f2) signals were found in hexaploid *A. densiflorus* 1. Scale bar = 10 µm
