# Supplementary material for: Karyological and nuclear DNA content variation of the genus Asparagus
Source: PLoS One. 2022 Mar 16;17(3):e0265405. doi: 10.1371/journal.pone.0265405 (PMC8926174; doi:10.1371/journal.pone.0265405)
Supplement: S3 Fig — The chromosomes were counterstained with DAPI (blue). Hexaploid A. densiflorus 2 (g3) has six 5S rDNA (g1) and six 45S rDNA (g2) signals. For tetraploid A. densiflorus 3 (h3) four 5S rDNA (h1) and four 45S rDNA (h2) signals were found. Hexaploid A. maritimus 1–3 (i3; j3; k3) has six 5S rDNA (i1; j1; k1) and twelve 45S rDNA signals (i2; j2; k2). For diploid A. officinalis ‘Darlise’ (l3) two 5S rDNA (l1; l4) and six 45S rDNA (l2; l5) signals were detected. Scale bar = 10 μm. (PDF) [file pone.0265405.s004.pdf]

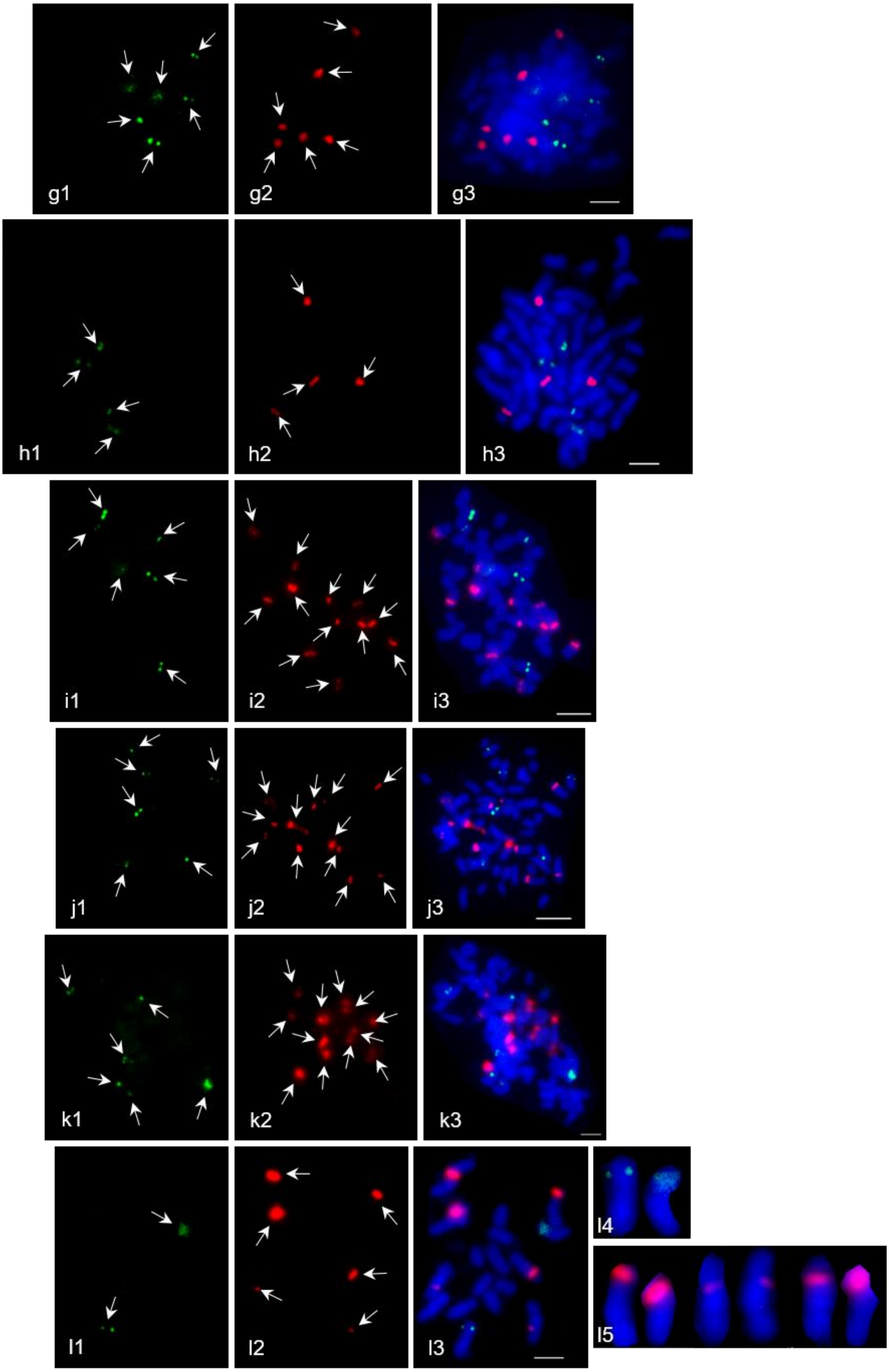

S3 Fig. FISH on mitotic metaphase spreads of *Asparagus* species using 5S rDNA (green) and 45S rDNA (red) as probes. The chromosomes were counterstained with DAPI (blue). Hexaploid *A. densiflorus* 2 (g3) has six 5S rDNA (g1) and six 45S rDNA (g2) signals. For tetraploid *A. densiflorus* 3 (h3) four 5S rDNA (h1) and four 45S rDNA (h2) signals were found. Hexaploid *A. maritimus* 1 -3 (i3; j3; k3) has six 5S rDNA (i1; j1; k1) and twelve 45S rDNA signals (i2; j2; k2). For diploid *A. officinalis* 'Darlise' (l3) two 5S rDNA (l1; l4) and six 45S rDNA (l2; l5) signals were detected. Scale bar = 10 µm
