# Supplementary material for: Karyological and nuclear DNA content variation of the genus Asparagus
Source: PLoS One. 2022 Mar 16;17(3):e0265405. doi: 10.1371/journal.pone.0265405 (PMC8926174; doi:10.1371/journal.pone.0265405)
Supplement: S4 Fig — The chromosomes were counterstained with DAPI (blue). Tetraploid A. officinalis `Steiners Violetta´ (m3) has four 5S rDNA (m1) and twelve 45S rDNA (m2) signals. Tetraploid A. pastorianus (n3) harbors four 5S rDNA (n1) and eight 45S rDNA signals. Two 5S rDNA (o1; o4) and eight 45S rDNA (o2; o4; o5) signals were detected for diploid A. plocamoides (o3). Diploid A. plumosus (p3) has two 5S rDNA (p1; p4) and two 45 S rDNA (p2; p5) signals. Four 5S rDNA (q1) and twelve 45s rDNA signals were found in tetraploid A. prostratus 1 (q3). Tetraploid A. prostratus 3 (r3) with four 5S rDNA (r1) and eight 45S rDNA signals (r2). Scale bar = 10 μm. (PDF) [file pone.0265405.s005.pdf]

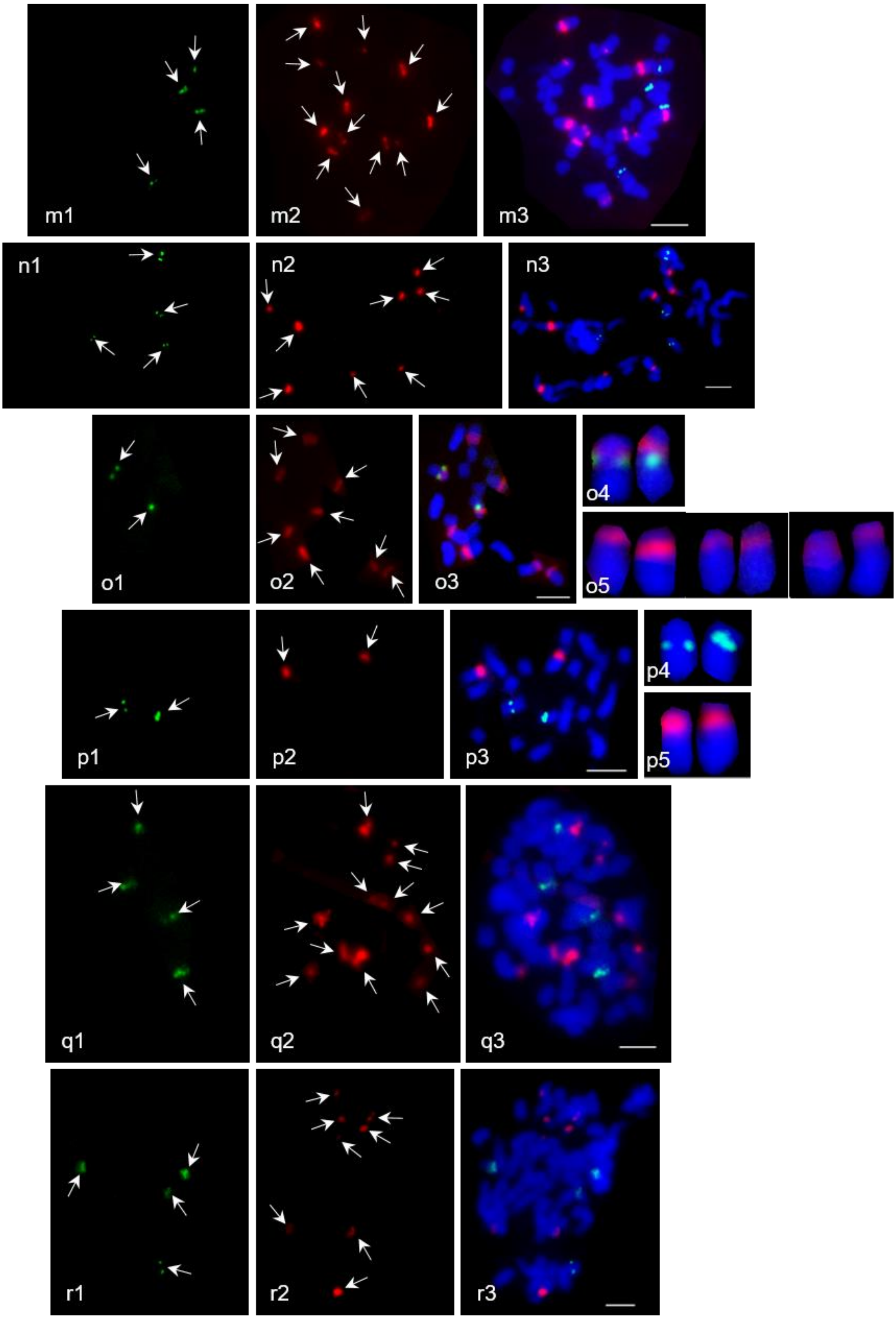

S4 Fig. FISH on mitotic metaphase spreads of *Asparagus* species using 5S rDNA (green) and 45S rDNA (red) as probes. The chromosomes were counterstained with DAPI (blue). Tetraploid *A. officinalis* `Steiners Violetta´ (m3) has four 5S rDNA (m1) and twelve 45S rDNA (m2) signals. Tetraploid *A. pastorianus* (n3) harbors four 5S rDNA (n1) and eight 45S rDNA signals. Two 5S rDNA (o1; o4) and eight 45S rDNA (o2; o4; o5) signals were detected for diploid *A. plocamoides* (o3). Diploid *A. plumosus* (p3) has two 5S rDNA (p1; p4) and two 45 S rDNA (p2; p5) signals. Four 5S rDNA (q1) and twelve 45s rDNA signals were found in tetraploid *A. prostratus* 1 (q3). Tetraploid *A. prostratus* 3 (r3) with four 5S rDNA (r1) and eight 45S rDNA signals (r2). Scale bar = 10 µm
