# Supplementary material for: Karyological and nuclear DNA content variation of the genus Asparagus
Source: PLoS One. 2022 Mar 16;17(3):e0265405. doi: 10.1371/journal.pone.0265405 (PMC8926174; doi:10.1371/journal.pone.0265405)
Supplement: S5 Fig — The chromosomes were counterstained with DAPI (blue). Tetraploid A. prostratus 4 (a3) harbors four 5S rDNA (a1) and eight 45S rDNA (a2) signals. Six 5S rDNA (b1) and twelve 45S rDNA signals were detected in hexaploid A. pseudoscaber (b3). Diploid A. ramosissimus (c3) has two 5S rDNA (c1; c4) and 45S rDNA (c2; c5) signals. Two 5S rDNA (d1; d4) and six 45S rDNA (d2; d5) signals were found in diploid A. scoparius (d3). Tetraploid A. setaceus (e3) has four 5S rDNA (e1) and sixteen 45S rDNA (e2) signals. Two 5S rDNA (f1; f4) and two 45S rDNA (f2; f5) signals were detected in diploid A. stipularis 1 (f3). Scale bar = 10 μm. (PDF) [file pone.0265405.s006.pdf]

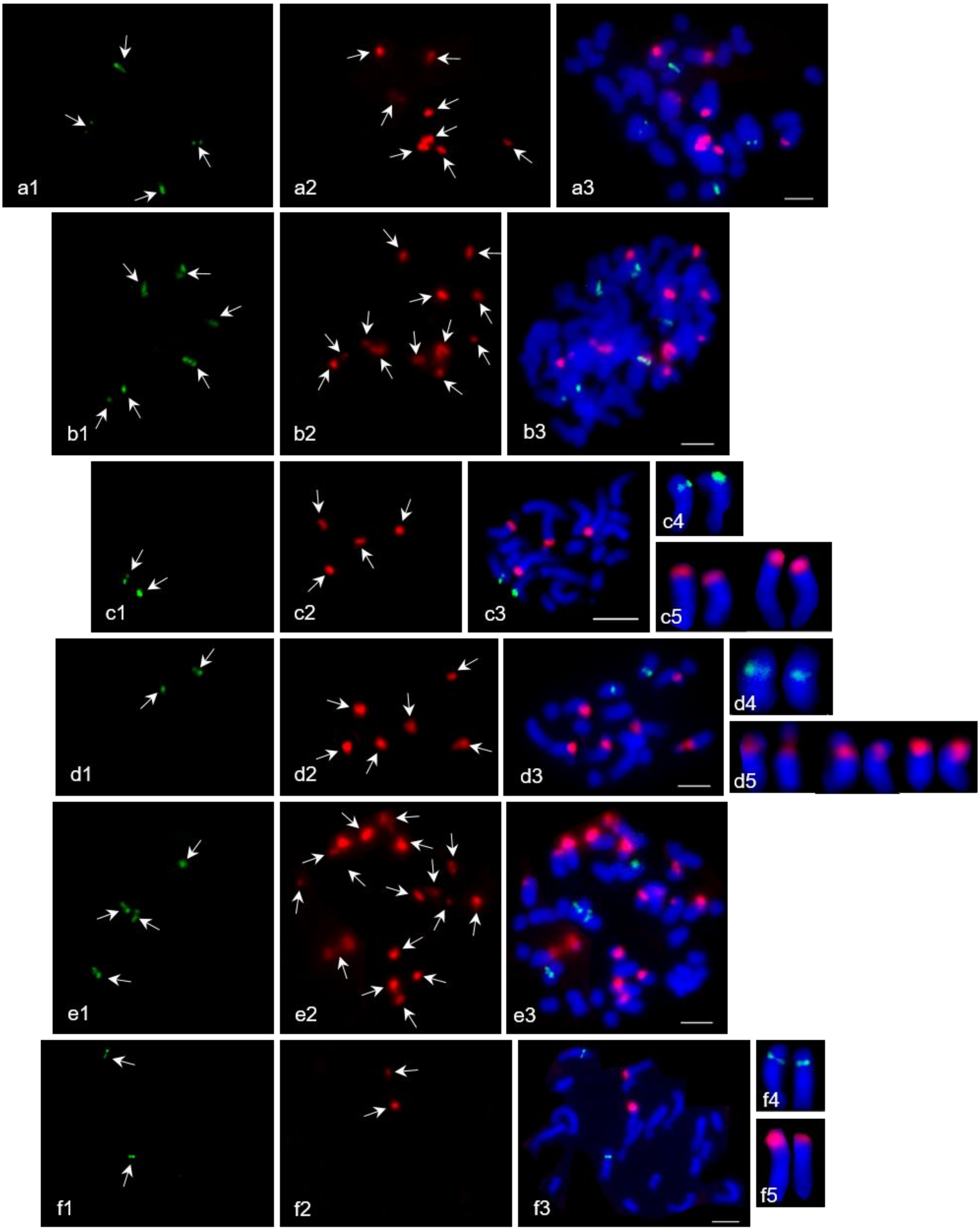


S5 Fig. FISH on mitotic metaphase spreads of *Asparagus* species using 5S rDNA (green) and 45S rDNA (red) as probes. The chromosomes were counterstained with DAPI (blue). Tetraploid *A. prostratus* 4 (a3) harbors four 5S rDNA (a1) and eight 45S rDNA (a2) signals. Six 5S rDNA (b1) and twelve 45S rDNA signals were detected in hexaploid *A. pseudoscaber* (b3). Diploid *A. ramosissimus* (c3) has two 5S rDNA (c1; c4) and 45S rDNA (c2; c5) signals. Two 5S rDNA (d1; d4) and six 45S rDNA (d2; d5) signals were found in diploid *A. scoparius* (d3). Tetraploid *A. setaceus* (e3) has four 5S rDNA (e1) and sixteen 45S rDNA (e2) signals. Two 5S rDNA (f1; f4) and two 45S rDNA (f2; f5) signals were detected in diploid *A. stipularis* 1 (f3). Scale bar = 10 µm
