# Supplementary material for: Karyological and nuclear DNA content variation of the genus Asparagus
Source: PLoS One. 2022 Mar 16;17(3):e0265405. doi: 10.1371/journal.pone.0265405 (PMC8926174; doi:10.1371/journal.pone.0265405)
Supplement: S6 Fig — The chromosomes were counterstained with DAPI (blue). Diploid A. stipularis 2–4 (g3; h3; i3) has two 5S rDNA (g1; g4; h1; i3; i4) and two 45S rDNA (g2; g5; h2; i2) signals. Four 5S rDNA (j1) and twelve 45S rDNA (j2) signals were detected for tetraploid A. verticillatus 1 (j3). In diploid A. verticillatus 3 (k3) two 5S rDNA (k1; k4) and four 45S rDNA (k2; k4; k5) signals were found. Diploid A. sp. (l3) has two 5S rDNA and four 45S rDNA signals. Scale bar = 10 μm. (PDF) [file pone.0265405.s007.pdf]

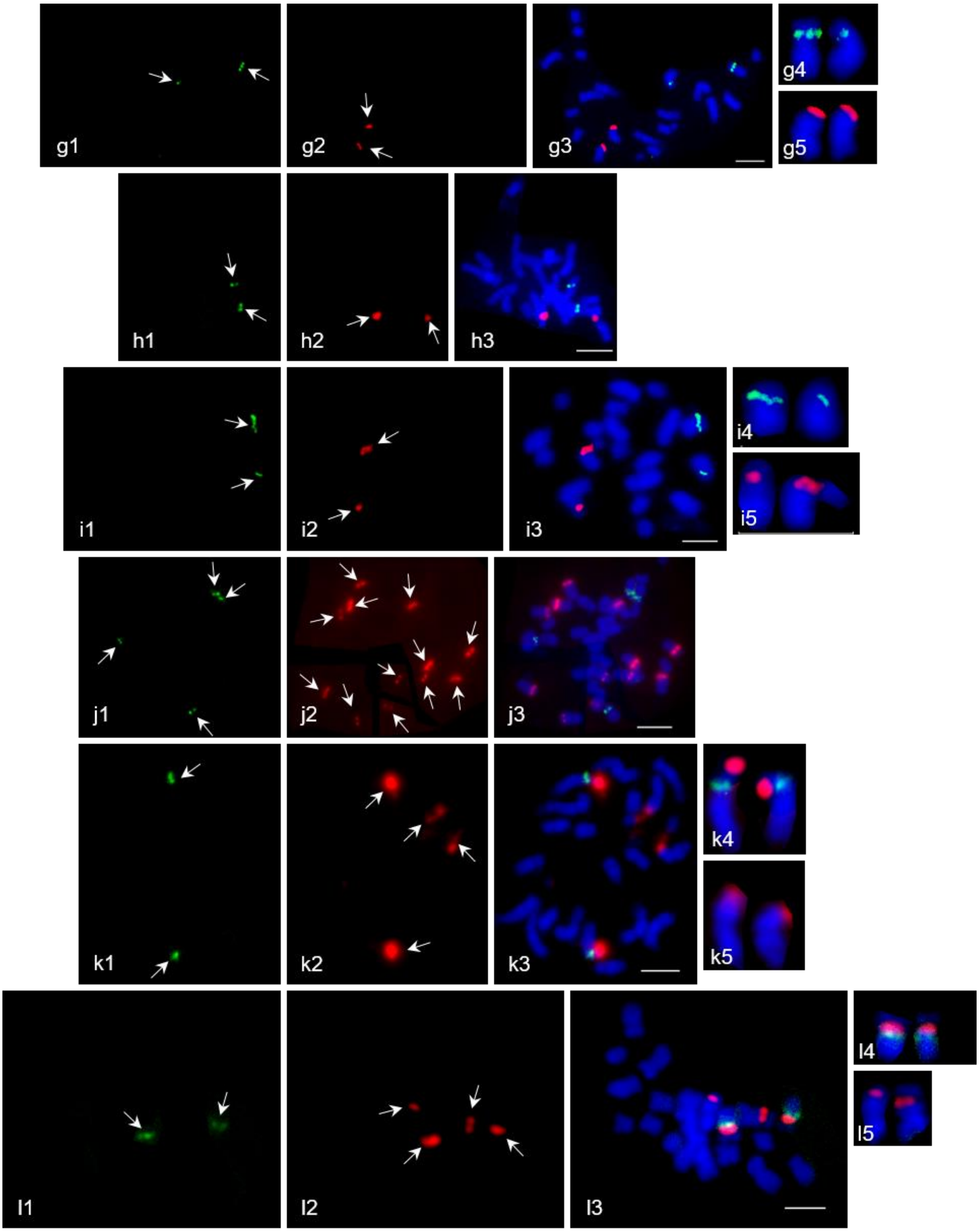

S6 Fig. FISH on mitotic metaphase spreads of *Asparagus* species using 5S rDNA (green) and 45S rDNA (red) as probes. The chromosomes were counterstained with DAPI (blue). Diploid *A. stipularis* 2 – 4 (g3; h3; i3) has two 5S rDNA (g1; g4; h1; i3; i4) and two 45S rDNA (g2; g5; h2; i2) signals. Four 5S rDNA (j1) and twelve 45S rDNA (j2) signals were detected for tetraploid *A. verticillatus* 1 (j3). In diploid *A. verticillatus* 3 (k3) two 5S rDNA (k1; k4) and four 45S rDNA (k2; k4; k5) signals were found. Diploid *A. sp.* (l3) has two 5S rDNA and four 45S rDNA signals. Scale bar = 10 µm
